# Percutaneous Fluoroscopy-guided Retrieval of a Fractured Pelvic Drain after Caesarean Section: A Case Report

**DOI:** 10.31729/jnma.8310

**Published:** 2023-10-31

**Authors:** Kamana Subba, Neil Gupta, Jacqueline Woodman, Vandana Dhingra

**Affiliations:** 1 Department of Obstetrics and Gynaecology, University Hospitals Coventry and Warwickshire NHS Trust, Coventry, United Kingdom; 2 Department of Interventional Radiology, University Hospitals Coventry and Warwickshire NHS Trust, Coventry, United Kingdom

**Keywords:** *case report*, *drain fragment retrieval*, *fractured pelvic drain*, *pelvic drain remnant*

## Abstract

Iatrogenic retention of surgical drains following drain entrapment and breakage is a never event and a preventable complication. The traditional approach for removing a fractured drain from the intra-peritoneal cavity involves exploratory laparotomy. However, over the last few decades, minimal access surgery has been a more popular retrieval method for retained surgical items from peritoneal and extraperitoneal cavities. We report a case of a 32-year-old woman with a fractured pelvic drain post-caesarean section. Postoperatively, the patient developed the signs of infection and features of bowel obstruction. The mechanical obstruction was ruled out by computed tomography scan. Multiple attempts were made to pull the pelvic drain out but the tube snapped, leaving about a quarter of its length. The drain remnant was retrieved using a non-invasive, inexpensive interventional radiology technique. We could not find any such report in the literature describing this innovative approach for retrieving a fractured pelvic drain.

## INTRODUCTION

The placement of a surgical drain during complex procedures for a prophylactic purpose is a common practice for most surgeons.^1^ However, difficulty in removal of the drain is an unusual encounter that carries the risk of drain fracture,^2^ which often necessitates surgical extraction. The first reported case of a fractured surgical drain was in 1973 by McCullough,^3^ which was retrieved through laparotomy. Traditionally, surgical re-exploration through open surgery has been the mainstay technique for retrieval of the severed retained drains from the abdominal and pelvic cavities.^4^ The complications can range from patients remaining completely asymptomatic^5^ to severe morbidity^6^ and mortality.^7^ We present a case report of a fractured pelvic drain retrieved through a percutaneous approach under fluoroscopic guidance.

## CASE REPORT

A 32-year-old woman in her fourth pregnancy underwent her fourth caesarean section at 38+6 weeks. The previous three caesarean deliveries were all carried out abroad with no surgical notes available. It was a challenging operation as intraoperatively, she was found to have a frozen pelvis with no plane to dissect where the omentum, peritoneum and uterus were fully plastered to the anterior abdominal wall. The procedure was completed with an inverted T incision on the skin and a classical uterine incision. A size 26 Ch silicone tube totalling 100 cm (Portex® Robinson Drainage System) was left in the pelvic cavity through a separate incision above the Joel-Cohen incision and secured with a silk suture. Total operative blood loss was 1000 ml with 250 ml RBC transfused back through the cell salvage.

On day 1 post-operative day, the patient developed abdominal distension, generalised tenderness, absent bowel sound, bilious vomiting, and high-grade pyrexia.

**Figure 1:**
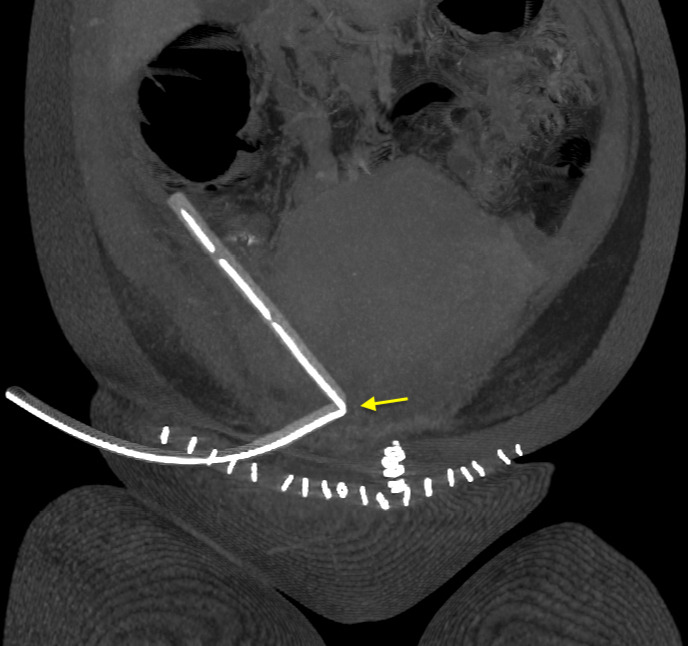
Coronal CT abdomen and pelvis showing Robinson drain on the right side of the pelvis positioned with the tip intraperitoneally in the right flank. A 90-degree angulation (arrow) in the surgical drain as it traverses through the rectus sheath.

On the same day, she had a CT scan of the abdomen and pelvis with contrast, which suggested likely colonic ileus and ruled out mechanical obstruction. The drain was reportedly on the pelvis's right side, with the tip intraperitoneally in the right flank adjacent to the colon (Figure 1).

She was commenced on intravenous antibiotics for suspected underlying infection and conservative measures for managing her ileus.

On day 2 post-operative day, she opened her bowels, and the patient remained clinically well. The drain had a minimal collection, and the plan was made to remove the drain the following day.

On day 3 post-operative day, the midwife and Senior House Officer (SHO) encountered difficulty removing the drain with failed attempts. The drain suture was taken out, but the drain was not moving with traction. This was escalated to the registrar, who was also unable to remove the drain by gentle traction. She then injected 40 ml of normal saline through the tube to release any possible intraluminal obstruction, and there was an easy flow of the fluid. The patient was provided with Entonox and moved to the left lateral position to relieve any pressure over the drain by pelvic organs. On a further attempt, the tube snapped, leaving about a quarter of its length. This case was then immediately escalated to the obstetric consultant. After extensive counselling, she was prepared to return to the theatre the other day for an exploratory laparotomy to retrieve the fractured pelvic drain.

On day 4 post-operative day, this case was discussed with the on-call Interventional Radiologist, who suggested he could try to retrieve the remaining tube instead of taking her back to the theatre for laparotomy. A fluoroscopy of the abdomen was performed, and the retained portion of the drain was removed successfully, as described below. The patient remained well and was discharged home the following day.

The skin staples were taken out on day 10 postoperative day. The patient was contacted at 6 months postpartum, and she reported having mild discomfort on the drain site, otherwise confirming an uneventful recovery.

**Description of the technique:** Informed consent was obtained, with risks of bleeding, infection, bowel perforation, peritonitis and failure discussed. The patient was positioned supine. Initially, no local anaesthetic was used, as this could obliterate the narrow cutaneous tract.

**Figure 2:**
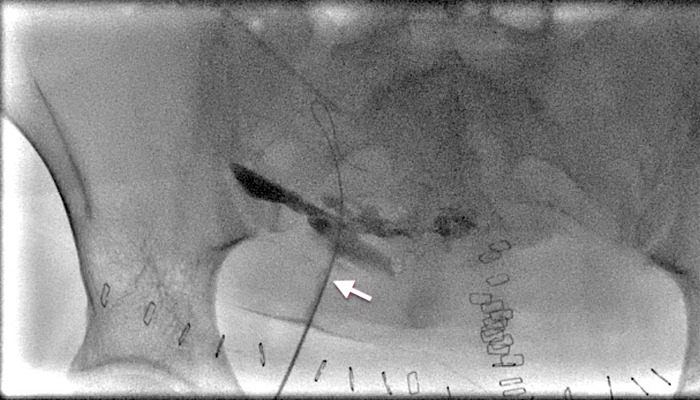
A 4 Fr Bolia catheter (arrow) with a looped Terumo guidewire entering the percutaneous tract towards the retained Robinson drain remnant.

A 4 Fr 20 cm Bolia catheter was inserted into the skin incision and a hydrophilic guidewire was inserted through the catheter. With gentle forward pressure on the catheter, the guide wire was advanced through the tract to form a loop which was then tracked through the lumen of the retained remnant of the drain (Figure 2).

The catheter was then advanced over the wire into the drain lumen, and the hydrophilic wire was exchanged for a stiff Amplatz wire. The Bolia catheter was removed and exchanged for a 23 cm long 7 Fr sheath. This enabled the placement of a second Amplatz wire to act as a "safety wire", and the 7 Fr sheath was removed.

**Figure 3:**
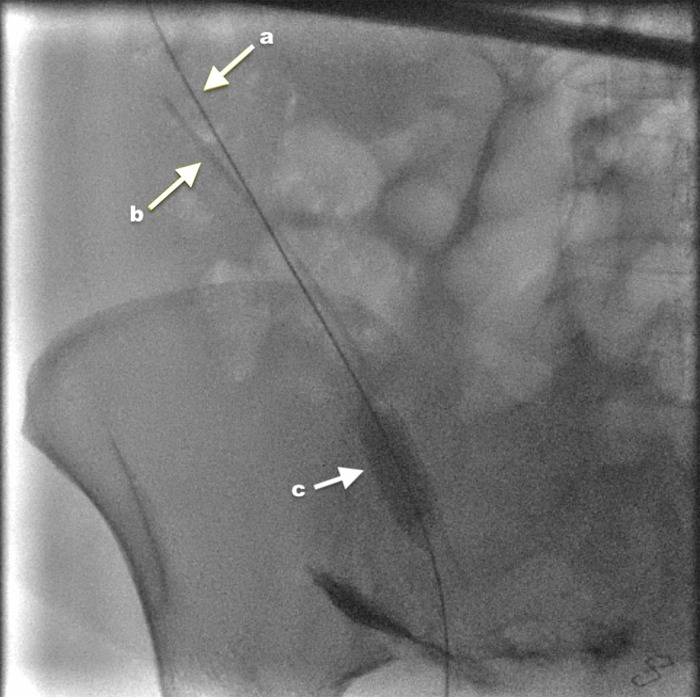
Two Amplatz wires (a and b) traversing the percutaneous tract and through the lumen of the drain remnant. A 12x20 mm balloon (c) is inflated within the lumen of the fractured drain to provide traction to withdraw it.

Initially, a single 12x20 mm standard angioplasty balloon was inserted over a wire and inflated within the lumen of the drain remnant (Figure 3).

**Figure 4:**
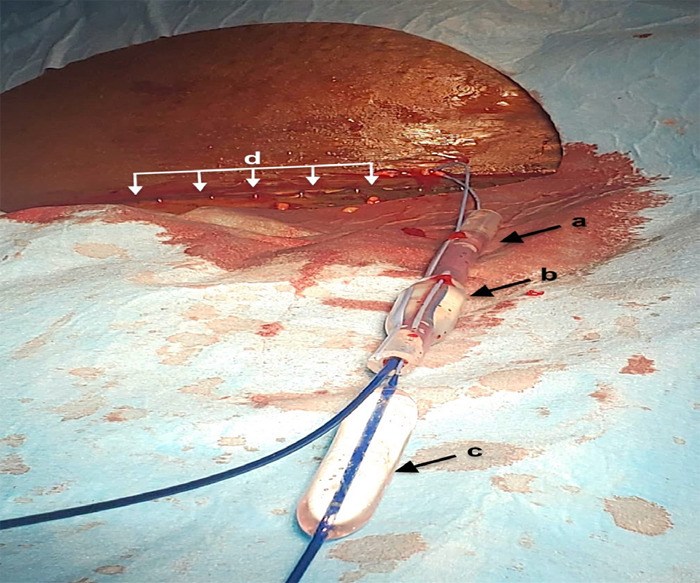
Successful removal of the drain remnant (a) showing two wires traversing through the lumen of the drain with a 12x20 mm balloon (b) inflated within the lumen of the drain and a 12x40 mm balloon (c) which was used to dilate the percutaneous tract, (d) Caesarean section wound.

This provided traction, but the drain could not be withdrawn through the narrow cutaneous tract and caused discomfort. A total of 10 ml 1% lignocaine was injected along the cutaneous tract, and Entonox was provided for pain relief so as not to impact breastfeeding. A 12x40 mm balloon was then inserted over the safety wire and inflated between the drain remnant and the cutaneous tract, then sequentially inflated and deflated along the tract whilst withdrawing the drain by maintaining traction on the 12x20 mm balloon. This enabled the successful "delivery" of the drain remnant (Figure 4).

## DISCUSSION

The leading cause for severed closed or open drains has been reported to be tethering by sutures that were placed to secure the drain in place.^8^ Appropriate techniques for placement and optimal management of drains will minimise complications related to these surgical implants.^9^ Fractured surgical items, such as drains,^10^ catheters,^11^ microcatheters,^12^ wires,^13^ broken devices,^14^ stents,^15^ have been reported to be successfully retrieved from various parts of the body through a variety of approaches. Since Kalies used the pan-endoscopic approach to remove retained Penrose drain in 1975,^16^ minimal access surgery has been a more popular retrieval method for retained surgical items from peritoneal and extraperitoneal cavities over the last few decades.^17^

The percutaneous approach under fluoroscopic guidance tends to be a procedure commonly performed by interventional radiologists for extracting foreign bodies in endovascular intervention^13^ and neuro intervention.^18^ Interventional radiology techniques are also getting widely popular amongst other specialities like urology,^10^ orthopaedics,^19^ etc.

In gynaecology, there are a few case reports of retrieval of retained drains from the pelvic cavity through traditional laparoscopic^17^ and transvaginal laparoscopic routes.^20^ Complications related to Robinson's drains have also been reported in the literature.^7^

Our case also depicts interventional radiological methods' potential and broader applicability in Obstetrics and Gynaecology. Various strategies can be employed during the drainage placement and securing^21^ to avoid potential issues during the retrieval of these surgical items. Direction and angulation of surgical drain tunnelling should always be considered to prevent the risk of kinking. The CT scan of the abdomen and pelvis of our patient clearly shows a 90-degree angulation in the surgical drain, worsened by the fact the angulation occurs as it traverses the rectus sheath. The rectus sheath and fascia are therefore acting as a "pinch point", causing a kink in the drain on traction and consequential snapping of the drain on forceful withdrawal.

Early referral to interventional radiology is essential as tract healing occurs quickly, and early intervention will result in a much higher foreign body retrieval rate.

If the drain remnant could not be initially canulated, alternative options include using a snare or crocodile biopsy forceps. No doubt, other equipment and techniques could also be adopted, but this simple and inexpensive technique proved successful and atraumatic, with very low risk.

Iatrogenic retention of surgical drains is a never event and a preventable complication. Every surgeon should have a good understanding of the functions of the various types of drains they use and exercise logical techniques while placing and removing these devices to avoid this medical error. This includes checking the tip of the drain after its removal and confirming the total length has been pulled out safely. The clinicians should maintain a high index of suspicion for early identification of breakage and retention of the fractured drain fragment before being deemed medically fit for discharge.
